# Mechanistic Analysis of Fisetin in Liver Diseases and Its Potential Therapeutic Application in IFALD—A Review of In Vitro and In Vivo Studies

**DOI:** 10.3390/nu18010102

**Published:** 2025-12-28

**Authors:** Marta Belka, Maciej Stawny, Michal M. Masternak, Violetta Krajka-Kuźniak

**Affiliations:** 1 Poznan University of Medical Sciences, Doctoral School, Bukowska 70, 60-812 Poznan, Poland; 2 Poznan University of Medical Sciences, Department of Pharmaceutical Biochemistry, Rokietnicka 3, 60-806 Poznan, Poland; vkrajka@ump.edu.pl; 3 Poznan University of Medical Sciences, Department of Pharmaceutical Chemistry, Rokietnicka 3, 60-806 Poznan, Poland; 4 Burnett School of Biomedical Sciences, University of Central Florida College of Medicine, Orlando, FL 32827, USA; 5 Poznan University of Medical Sciences, Department of Head and Neck Surgery, 61-701 Poznan, Poland

**Keywords:** fisetin, signaling pathways, liver diseases, IFALD

## Abstract

Fisetin (3,3′,4′,7-tetrahydroxyflavone) is a naturally occurring flavonol in fruits and vegetables. It exhibits diverse biological activities, including anti-inflammatory, antioxidant, senolytic, and lipid-lowering properties. This review explores the molecular mechanisms underlying fisetin’s hepatoprotective effects and evaluates its potential application in Intestinal Failure-Associated Liver Disease (IFALD), a severe complication associated with total parenteral nutrition (TPN). IFALD is characterized by inflammation, cholestasis, steatosis, oxidative stress, and dysregulated lipid and bile acid metabolism. Fisetin modulates several key signaling pathways, including NF-κB, Nrf2, AMPK, and SIRT1, leading to reduced inflammatory cytokine expression, enhanced antioxidant defenses, and improved lipid homeostasis. Fisetin shows potential anti-fibrotic and microbiota-modulating effects. More importantly, fisetin is recognized as a potent senolytic agent, selectively activating pro-apoptotic pathways in senescent cells, which are known sources of inflammation and tissue damage. However, despite its promising pharmacological profile, the poor bioavailability of fisetin remains a significant limitation, particularly for parenteral use. Emerging drug delivery systems such as liposomes and nanoparticles offer potential solutions. Given its broad spectrum of beneficial effects and favorable safety profile, fisetin represents a compelling candidate for future studies in the prevention and management of IFALD.

## 1. Introduction

Fisetin is a naturally occurring flavonol that has attracted increasing attention due to its diverse biological activities, including anti-inflammatory, antioxidant, neuroprotective, and senolytic properties [1]. Recent reviews and experimental studies published within the past three years have expanded the understanding of fisetin’s biological properties, highlighting its anti-inflammatory, antioxidant, metabolic, and senotherapeutic potential [2,3,4]. In particular, recent work has demonstrated that fisetin improves insulin sensitivity, reduces hepatic lipid accumulation, modulates redox signaling, and attenuates inflammatory responses in metabolic liver disorders [5,6]. These new findings strengthen the rationale for investigating fisetin as a therapeutic candidate in liver diseases with inflammatory and metabolic components.

The pleiotropic biological actions of fisetin are attributable to its ability to modulate multiple conserved cellular pathways. Its antioxidant effects arise from direct radical-scavenging activity mediated by hydroxyl groups on the flavone backbone, as well as indirect antioxidant responses through activation of the Nrf2 pathway [7,8]. Fisetin’s anti-inflammatory properties are driven mainly by suppression of NF-κB signaling and inhibition of NLRP3 inflammasome activation [9]. Neuroprotective effects result from its capacity to reduce ROS accumulation, stabilize mitochondrial function, and modulate ERK1/2 and PI3K/Akt pathways [10]. Importantly, fisetin exerts senolytic activity by selectively inducing apoptosis in senescent cells through coordinated inhibition of NF-κB and activation of p53, thereby reducing SASP secretion [3].

Additionally, fisetin has shown promise in a variety of other liver pathologies, including non-alcoholic fatty liver disease (NAFLD) [11,12,13,14], alcoholic liver disease (ALD) [5,6], drug-induced liver injury (DILI) [15,16,17], and hepatic fibrosis [11], further supporting its broad hepatoprotective profile. In the following sections, we will discuss how these diverse mechanisms of fisetin intersect with the pathophysiological features of Intestinal Failure-Associated Liver Disease (IFALD), emphasizing its potential therapeutic relevance in this specific liver condition. IFALD is a form of liver dysfunction associated with total parenteral nutrition (TPN) administration. Typically developing after approximately two weeks of TPN, IFALD affects nearly half of adult patients undergoing prolonged parenteral nutrition [18]. It is characterized by inflammation, cholestasis, hepatic steatosis, and fibrosis, frequently accompanied by dyslipidemia and impaired bile acid homeostasis [19]. Clinically, IFALD manifests through elevated serum levels of alanine aminotransferase (ALT), aspartate aminotransferase (AST), alkaline phosphatase (ALP), and total bilirubin [20]. Numerous strategies have been explored to mitigate IFALD, including modifications to lipid emulsions (e.g., fish oil-based formulations) [21], bile acid modulators, and the administration of antibiotics [21,22]. Although several preventive and therapeutic strategies are currently tested in clinical practice—including UDCA, optimized lipid emulsions, and infection management—none provide consistently effective or comprehensive protection against IFALD. Therefore, the condition still lacks a definitive, broadly effective therapy.

This review aims to evaluate the role of fisetin in the context of liver diseases with inflammatory and metabolic components and assess its potential therapeutic application in preventing and treating IFALD.

## 2. Fisetin Overview

Fisetin (3,3′,4′,7-tetrahydroxyflavone) is a naturally occurring flavonol in various fruits and vegetables, including apples, strawberries, grapes, tomatoes, onions, and cucumbers. Among these, strawberries are considered one of the richest natural sources, containing up to 160 μg/g of fisetin [1,23]. Its widespread presence in edible plants contributes to its accessibility as a dietary supplement and a subject of pharmacological interest.

Chemically, fisetin is characterized by a flavone backbone with four hydroxyl groups at positions 3, 7, 3′, and 4′. Its molecular formula is C_15_H_10_O_6_, and its structure consists of two benzene rings (A and B) connected via a three-carbon bridge, forming a closed pyrone ring (C). The hydroxyl groups enhance its antioxidant properties by enabling free radical scavenging and metal ion chelation [24,25]. This structure also facilitates interactions with various cellular targets, which underlie many of fisetin’s biological activities [1].

Fisetin has attracted increasing attention due to its diverse biological properties, including anti-inflammatory, antioxidant [24], neuroprotective [10], anti-aging [3], and anti-cancer effects [26]. Despite these promising activities, its clinical utility is limited by poor water solubility, high lipophilicity, and low oral bioavailability. After oral administration, fisetin is rapidly metabolized through phase II processes, primarily via glucuronidation and sulfation in the liver and intestines, resulting in the predominance of conjugated metabolites in plasma [27].

Notably, fisetin is absorbed in the gastrointestinal tract and can cross the blood–brain barrier, making it an attractive candidate for neuroprotective therapies. However, its limited systemic availability has prompted the development of various formulation strategies to enhance absorption and biological efficacy, including fisetin-loaded nanoparticles, liposomes, and inclusion complexes with cyclodextrins [23]. These delivery systems improve solubility, protect fisetin from metabolic degradation, and facilitate more efficient transport across biological membranes [1]. While the mechanisms governing fisetin’s cellular uptake and distribution are not fully understood, they likely involve interactions with specific transporter proteins [10].

Preclinical toxicological studies suggest that fisetin has a favorable safety profile. Animal studies have shown low acute toxicity and minimal adverse effects at therapeutically relevant doses. Higher doses may cause gastrointestinal discomfort or hepatic stress, though these effects have been observed mainly in rodent models [3].

Preclinical studies indicate that fisetin has a favorable safety profile. In rodent models, oral doses ranging from 10 to 100 mg/kg/day and intraperitoneal doses of 10 to 40 mg/kg were well tolerated, with minimal adverse effects [5,6,15,16,17,28]. Human supplementation studies typically employ oral doses between 20 and 100 mg/day, with ongoing clinical trials investigating senolytic activity using intermittent high-dose regimens (e.g., 20 mg/kg/day for 2–3 days) [4]. Reported adverse effects are rare and generally limited to mild gastrointestinal discomfort. However, higher parenteral doses may increase hepatic metabolism and transient hepatocellular stress, underscoring the need for optimized formulation strategies for intravenous delivery.

Fisetin has not demonstrated mutagenic, genotoxic, or carcinogenic properties in standard laboratory assays. In vitro research further supports its safety, showing no significant cytotoxicity in non-cancerous cell lines at physiologically relevant concentrations. Nonetheless, fisetin may exhibit pro-oxidant behavior at higher doses or alter the activity of drug-metabolizing enzymes, suggesting potential interactions with other compounds. Importantly, recent studies have indicated that fisetin successfully targets some senescent cells in humans, particularly reducing the number of senescent peripheral blood mononuclear cells and thereby reducing levels of senescent-associated secretory phenotypes (SASP) [29]. There are also several pending clinical trials for a variety of human diseases [4]. However, there is a continuous need to improve understanding of the detailed mechanism of action, as well as to enhance bioavailability and stability during treatment.

The extensive attention in basic and clinical research suggests that fisetin shows strong potential as a nutraceutical and therapeutic agent. However, further comprehensive studies, particularly in humans and sensitive populations such as pregnant individuals or those with liver dysfunction, are essential to fully establish its long-term safety and tolerability [30].

## 3. Methodology

A literature review was conducted to summarize and critically discuss current evidence on the effects of fisetin in the context of liver function and hepatic pathology. The literature search was performed on 13 January 2025, using the PubMed and Scopus databases. In PubMed, all available fields were searched, whereas in Scopus the search was limited to titles, abstracts, and keywords. The search strategy included combinations of the following terms: “fisetin” AND “liver”, “fisetin” AND “hepatotoxicity”, “fisetin” AND “liver injury”, “fisetin” AND “cholestasis”, as well as terms related to inflammation and oxidative stress.

The search was restricted to original research articles published in English between 2015 and 2025. Studies were considered eligible if they investigated fisetin as a single bioactive compound in relation to hepatoprotection, liver injury, or liver-related molecular and cellular mechanisms, and if they reported measurable biochemical, molecular, or histological outcomes. Review articles, conference abstracts, commentaries, and studies in which fisetin was administered in combination with other compounds without the possibility of isolating its individual effects were excluded. Additionally, studies that did not directly address hepatic mechanisms or focused primarily on hepatic ischemia and reperfusion injury, with limited mechanistic relevance to the scope of this review, were not considered.

**Figure 1:**
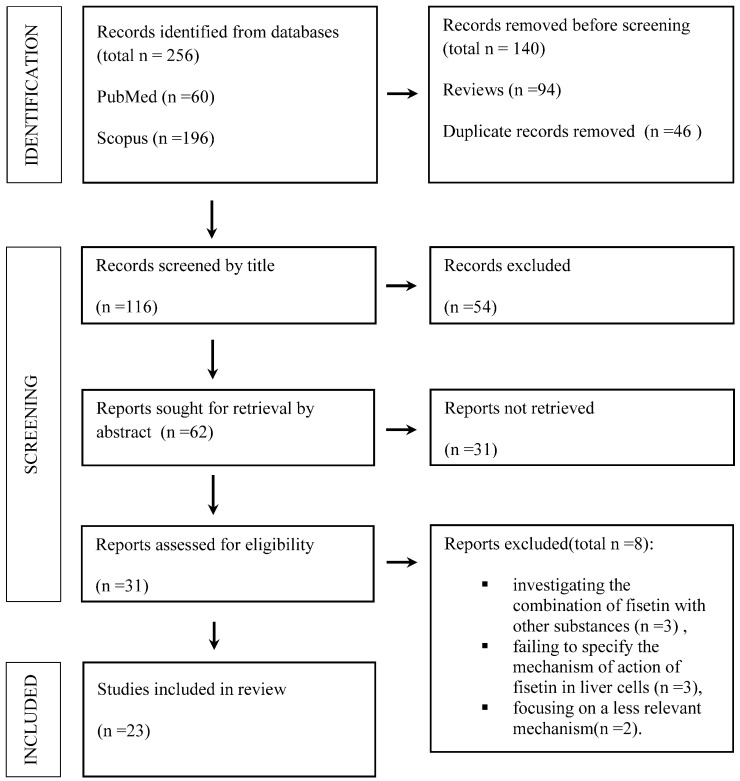
A flowchart showing the results of screening and literature searches.

A total of 256 records were retrieved from the initial database search (60 from PubMed and 196 from Scopus). After the removal of duplicate entries and non-eligible publication types, the remaining studies were examined for relevance. Screening of titles and abstracts allowed for the exclusion of clearly unrelated articles, and a subset of publications was subsequently assessed in full text. During full-text evaluation, several studies were excluded because fisetin was administered in combination with other agents, the hepatic mechanism of action was not sufficiently defined, or the experimental focus was limited to ischemia–reperfusion injury with marginal relevance to the objectives of this review. An overview of the literature search and study selection process is summarized in Figure 1.

## 4. Molecular Basis of Fisetin’s Hepatoprotective Activity

Fisetin exhibits a multifaceted hepatoprotective profile, targeting several key pathological processes associated with liver injury (Table 1).

### 4.1. Inhibition of NF-κB and Inflammatory Signaling

Fisetin inhibits the NF-κB signaling pathway, a central regulator of inflammation. It downregulates the expression of pro-inflammatory cytokines such as TNF-α, IL-6, IL-1β, and IL-18 [31,34,37,38,40], and reduces levels of cyclooxygenase-2 (COX-2) [34]. Additionally, fisetin interferes with the activation of the NLRP3 inflammasome complex, further mitigating hepatic inflammatory responses [39,41].

### 4.2. Senolytic Action Through Induced Apoptosis

In response to intra- or extracellular stress, cells can enter a state of senescence, recognized as a stable cell cycle arrest through upregulation of cyclin-dependent kinase inhibitors including p16^INK4a^, p21^CIP1/WAF1^, and p15^INK4b^. Despite cell cycle arrest, senescent cells produce and secrete SASP, composed mainly of inflammatory cytokines, including IL-6, IL-1, IL-8, TNF-α, PAI-1, and CXCL-2. Fisetin exhibits senolytic activity by inducing apoptosis through various signaling pathways, but in vitro studies showed that it targets senescent cells selectively with no pro-apoptotic action in non-senescent, healthy cells [44]. It was shown that in senescent cells, fisetin simultaneously inhibits the NF-κB pathway and activates the p53 signaling pathway. The other signaling pathways associated with apoptosis induced by fisetin also involve inhibition of HSF1 activity, ERK1/2 activation, and targeting the PI3K pathway. Previous rodent studies showed that the senolytic action of fisetin improves lifespan and healthspan in mice [44].

### 4.3. Activation of Nrf2 and Antioxidant Defense

Fisetin activates the Nrf2 (nuclear factor erythroid 2–related factor 2) pathway [39], leading to increased expression of antioxidant enzymes including heme oxygenase-1 (HO-1) [43], superoxide dismutase (SOD), NAD(P)H: quinone oxidoreductase 1 (NQO1) [36], and glutathione-related enzymes (GPX1, GSR, GSH) [32,33,34]. This enhances the liver’s capacity to counteract oxidative stress and maintain redox homeostasis.

### 4.4. Regulation of Lipid Metabolism via AMPK, SIRT1, and PPAR Pathways

Fisetin regulates lipid homeostasis by activating AMP-activated protein kinase (AMPK) [42] and sirtuin 1 (SIRT1). These pathways suppress lipogenic transcription factors such as sterol regulatory element-binding protein-1C (SREBP-1C) and enzymes like stearoyl-CoA desaturase 1 (SCD1) [12,28]. Concurrently, fisetin promotes fatty acid β-oxidation via upregulation of PPARα and reduces cholesterol biosynthesis by inhibiting HMG-CoA reductase (HMGCR) and Acyl-CoA: Cholesterol Acyltransferase (ACAT) [11].

### 4.5. Anti-Fibrotic Action Through TGF-β Pathway Suppression

Fisetin attenuates fibrotic progression by downregulating transforming growth factor-beta 1 (TGF-β1) and associated fibrosis markers, including alpha-smooth muscle actin (α-SMA), matrix metalloproteinases (MMP-2, MMP-9), and their inhibitors. These effects limit extracellular matrix deposition and hepatic scarring [5,16].

### 4.6. Modulation of Gut Microbiota Composition

Fisetin positively influences the gut microbiome by enhancing the abundance of beneficial microbial species, such as *Akkermansia muciniphila* and *Bifidobacterium breve*. These changes contribute to improved gut barrier function and reduced translocation of endotoxins like lipopolysaccharides (LPS), alleviating liver inflammation and promoting systemic metabolic health [35]. Together, these mechanisms establish fisetin as a promising candidate for treating liver diseases characterized by inflammation, oxidative stress, dyslipidemia, and fibrosis.

The following section will contextualize these molecular actions within the complex pathogenesis of IFALD, with particular attention to the role of reactive oxygen species (ROS), mitochondrial stress, and impaired antioxidant defenses—hallmarks of parenteral nutrition-induced liver injury.

Beyond the mechanisms summarized above, several recent studies further support fisetin’s hepatoprotective potential. Fisetin has been shown to suppress ER stress by reducing GRP78 signaling and restoring mitochondrial membrane potential [36]. It also regulates autophagy, a key survival pathway in hepatotoxic injury, by promoting LC3-II conversion and inhibiting mTOR activation in APAP-induced liver damage models [17]. Additionally, fisetin attenuates hepatocyte apoptosis by modulating the Bcl-2/Bax ratio and caspase-3 activity, thereby limiting parenchymal cell loss [34]. Together, these mechanisms highlight the broad spectrum of cytoprotective effects of fisetin in hepatic injury.

In addition, fisetin influences several interconnected signaling networks that collectively contribute to hepatoprotection. Beyond inhibiting NF-κB activation by suppressing IKKβ phosphorylation and stabilizing IκBα, fisetin enhances the nuclear translocation of Nrf2 by preventing Keap1-mediated degradation. Through AMPK and SIRT1 activation, increase the AMP/ATP ratio, promotes fatty acid β-oxidation, and suppresses lipogenesis. Moreover, by modulating p53–p21 signaling and reducing SASP-related cytokines, fisetin exhibits senolytic properties that may alleviate chronic inflammation within hepatic tissue. These complementary actions underline the multifactorial nature of fisetin’s hepatoprotective profile.

## 5. Pathomechanism of IFALD

The pathological mechanisms underlying IFALD are complex and multifactorial. Several factors contribute to its development, including phytosterols, central line-associated bloodstream infections (CLABSIs), gut dysbiosis, oxidative stress, and prolonged inflammation (Table 2).

Phytosterols, present in the vegetable oils (primarily soybean oil) used in parenteral nutrition (PN) emulsions, are a key PN-dependent factor. These compounds structurally resemble cholesterol and disrupt its metabolism by inhibiting the liver’s farnesoid X receptor (FXR), leading to increased production of bile acids and triglycerides. Excess bile acids are usually exported via hepatic bile transporters into the intestinal lumen, where they activate intestinal FXR, triggering a negative feedback loop (mediated by FGF19 and FGFR4) that suppresses further bile acid synthesis [45]. However, in the presence of LPS, a second important factor, this balance is disturbed.

LPS induces liver inflammation by binding to TLR4 receptors on Kupffer cells, activating the NF-κB pathway, and promoting the release of pro-inflammatory cytokines. Inflammatory conditions impair the function of bile acid transporters, hindering bile acid efflux from the liver, disrupting the negative feedback mechanism, and contributing to intrahepatic cholestasis. LPS can originate from bloodstream infections associated with central venous catheters or gut dysbiosis, both of which are common in patients receiving PN. Reduced or absent enteral feeding promotes gut microbial imbalances, favoring the overgrowth of Gram-negative bacteria, such as *Bacteroidaceae* and *Enterobacteriaceae*, which further increases endotoxin production and hepatic injury. Prolonged PN use is associated with elevated levels of pro-inflammatory cytokines, including TNF-α, IL-6, and the profibrotic factor TGF-β [46]. The lipid emulsions used in PN, particularly those rich in omega-6 fatty acids, contribute significantly to this inflammatory state. Omega-6 fatty acids are precursors for pro-inflammatory eicosanoids and can directly stimulate cytokine production. Furthermore, omega-6-rich emulsions may mimic LPS activity by activating the TLR4 signaling pathway [47]. Studies have demonstrated that LPS stimulation downregulates FXR mRNA levels in the liver [51], removing its normal suppressive effect on NF-κB and thereby amplifying inflammation [52]. Higher levels of TNF-α and IL-6 have been documented in animals receiving PN [19], reinforcing the link between PN and liver inflammation. Oxidative stress is another central mechanism in IFALD pathogenesis. In the setting of TPN, excessive ROS production overwhelms the hepatocyte antioxidant defenses, leading to mitochondrial dysfunction and endoplasmic reticulum (ER) stress. Lipid-rich PN formulations, particularly those high in omega-6 fatty acids, exacerbate oxidative stress by promoting lipid peroxidation and activating pro-inflammatory pathways.

Studies have shown that PN reduces the expression of genes involved in antioxidant defense, including *Gstp1*, *Gstm1*, *Nqo1*, *Ho-1*, and *Gclc*, in mice receiving Intralipid-based emulsions [48]. In neonatal pig models, TPN was associated with GPX1 hypermethylation and decreased mRNA expression of *GPX1*, *GCLC*, and *galactosylceramide sulfotransferase (GSase).* In contrast, supplementation with glutathione (GSH) prevented these changes and enhanced the expression of Nrf2 and SOD2 [49]. These findings suggest that PN-induced oxidative stress results from excessive nutrient infusion and suppression of endogenous antioxidant systems, contributing to hepatic steatosis, cholestasis, and fibrosis. Parenteral nutrition also disrupts lipid and bile acid metabolism. PN-fed animals exhibit increased hepatic lipid accumulation, which is associated with the upregulated expression of lipogenic genes such as *SREBP-1C*, *FAS*, *LPL*, and *SCD1* [50]. Persistent inflammation and oxidative stress further drive the progression from steatosis to liver fibrosis. Elevated levels of TGF-β and MMP-9 in the context of IFALD contribute to extracellular matrix remodeling and fibrogenesis, ultimately leading to irreversible liver damage [53].

## 6. The Potential Role of Fisetin in Preventing and Treating IFALD

As noted in the Introduction Section, treatment strategies for IFALD are limited. Many strategies for preventing and treating IFALD have been explored, and these generally involve modifying the PN regimen. Such modifications include adjusting the duration of nutrition and changing the composition of the PN solution, often by incorporating fish oil-based lipid emulsions.

However, none of these nutritional strategies are considered universally effective or sufficient on their own. Pharmacological therapies, such as ursodeoxycholic acid (UDCA) or ursodiol, phenobarbital, metronidazole, erythromycin, and cholecystokinin-octapeptide, have also been investigated. Although these drugs were tested in various clinical trials, none are currently established or widely used as standard pharmacological treatments for IFALD [54,55].

IFALD involves chronic activation of inflammatory pathways, notably via NF-κB and pro-inflammatory cytokines, including TNF-α and IL-6 [46,51]. Fisetin mitigates this response by suppressing NF-κB activation and the subsequent expression of downstream cytokines. Its additional inhibition of TGF-β signaling suggests a role in halting or reversing fibrosis. The particular advantage of fisetin lies in its ability to simultaneously target the inflammatory, oxidative, metabolic, and fibrotic components of IFALD. This multimodal approach is particularly relevant given the multifactorial pathogenesis of the disease, in which interventions targeting a single mechanism often fail to provide sufficient clinical benefit.

Central to this pathology is oxidative stress, driven by the excessive production of ROS. TPN formulations rich in omega-6 fatty acids exacerbate ROS generation and lipid peroxidation, overwhelming hepatic antioxidant defenses and inducing mitochondrial and ER stress. Fisetin counters these effects by activating the Nrf2 pathway, which enhances the expression of antioxidant enzymes such as HO-1, SOD, GPX1, and glutathione-related proteins. This response restores redox balance and protects hepatocytes from ROS-mediated injury, potentially slowing the progression toward cholestasis and fibrosis.

**Figure 2:**
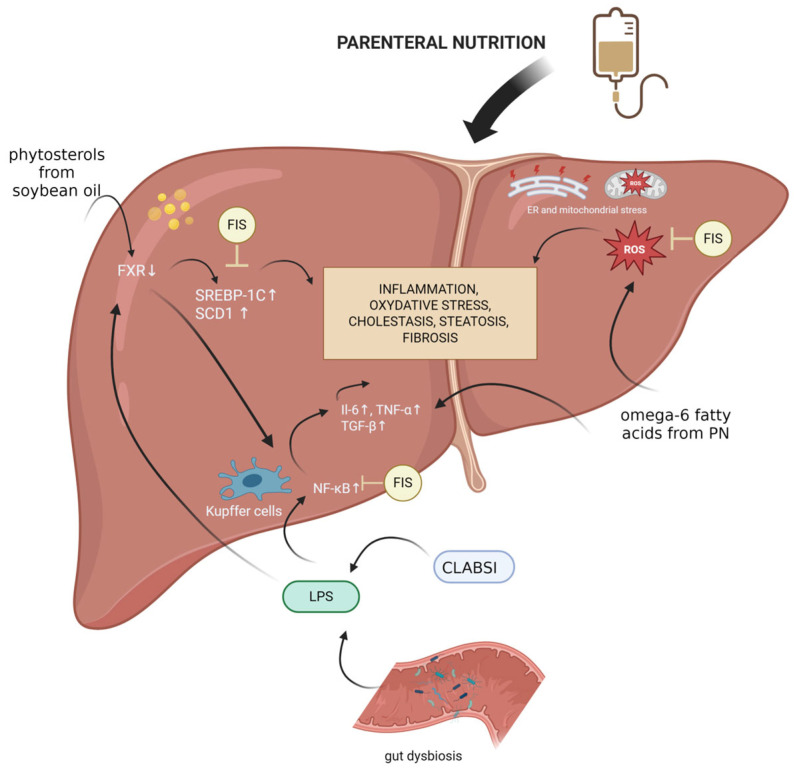
Mechanisms underlying IFALD development and fisetin’s potential protective effects. Created with BioRender.com. Abbreviations: CLABSI, central line-associated bloodstream infection; ER, endoplasmic reticulum; FIS, fisetin; FXR, farnesoid X receptor; IL-6, interleukin-6; LPS, lipopolysaccharide; PN, parenteral nutrition; ROS, reactive oxygen species; SCD1, stearyl-coenzyme A desaturase 1; SREBP-1C, Sterol Regulatory Element-Binding Protein 1c; TGF-β, transforming growth factor beta; TNF-α, tumor necrosis factor.

Dysregulated bile acid metabolism, a hallmark of IFALD, results partly from phytosterol-mediated suppression of FXR signaling. While fisetin does not directly activate FXR [56], it modulates downstream metabolic pathways, such as AMPK activation and SREBP inhibition, thereby supporting bile acid and lipid homeostasis. This indirect modulation may offer therapeutic benefits in cases where FXR signaling is impaired (Figure 2).

Moreover, fisetin’s influence on the gut–liver axis is of growing interest. By promoting the growth of beneficial microbial species and enhancing gut barrier integrity, fisetin may reduce endotoxin translocation and Kupffer cell activation—important contributors to systemic and hepatic inflammation during TPN. Additionally, by just removing an excess of senescent cells from either the gut or the liver tissue, Fisetin can promote a favorable environment for improved gut biosis and a healthier liver due to suppressed SASP secretion within these two organs.

Ursodeoxycholic acid (UDCA), a natural hydrophilic bile acid, has been investigated for conditions such as cholestatic liver disease and IFALD. While UDCA has shown some beneficial effects in IFALD, robust evidence supporting its efficacy in this setting is still limited. Its primary actions involve reducing the toxicity of the bile acid pool and exerting cytoprotective effects, partly through inhibition of apoptosis [57,58]. In comparison, fisetin appears to have a more favorable safety profile and a broader spectrum of pleiotropic activities, including pronounced antioxidant and anti-inflammatory effects, which makes it a particularly attractive candidate for further investigation relative to UDCA. Compared with existing therapeutic approaches such as UDCA or fish oil-based lipid emulsions, fisetin offers a wider mechanistic profile, combining anti-inflammatory, antioxidant, metabolic, anti-fibrotic, and senolytic effects that may provide complementary or enhanced benefits in the multifactorial context of IFALD.

These pleiotropic actions suggest that fisetin may complement or even surpass existing therapies, particularly given the multifactorial pathogenesis of IFALD.

Fisetin’s antioxidant, anti-inflammatory, anti-fibrotic, and metabolic regulatory actions collectively address key pathogenic drivers of IFALD, highlighting its potential as a multi-target therapeutic agent in this challenging clinical setting.

## 7. Consideration of Supplying Fisetin to Patients Fed Parenterally

Many studies on fisetin have primarily focused on its oral administration. Compared to oral administration, fisetin exhibits different behavior when administered intravenously. Following an intravenous administration of 10 mg/kg in rats, fisetin is rapidly biotransformed in the liver into fisetin sulfates through conjugation. In contrast, following oral administration at a dose of 50 mg/kg, a portion of fisetin remains unmetabolized in the systemic circulation. This suggests that fisetin undergoes less sulfation in the intestinal enterocytes than in hepatocytes, with glucuronidation being the dominant pathway after oral intake [27].

In cancer studies involving mice, intravenous administration of a fisetin nanoemulsion showed relatively higher toxicity. However, compared to intraperitoneal administration of free fisetin, the nanoemulsion significantly increased plasma concentrations of the compound, indicating improved bioavailability through this method [59].

According to data retrieved from the ClinicalTrials.gov registry, current clinical trials involving fisetin include 20 interventional studies focused primarily on its senolytic, anti-inflammatory, and functional health-supporting properties. Most trials investigate healthy aging, frailty, and multimorbidity in older adults, assessing whether fisetin can reduce the burden of senescent cells, improve mobility, decrease inflammation, and enhance overall physical function (e.g., NCT07195318, NCT06431932, NCT03675724). Additional studies explore its use in vascular conditions such as peripheral arterial disease and endothelial dysfunction, as well as in rehabilitation following cancer treatment, particularly among breast cancer survivors, where fisetin is evaluated for its potential to reduce fatigue and support functional recovery. Fisetin is also being investigated in orthopedic conditions, including carpal tunnel syndrome and knee osteoarthritis, as well as in several COVID-19 studies examining its ability to mitigate inflammatory complications in older adults. Overall, current clinical trials on fisetin focus predominantly on aging-related conditions, inflammation, rehabilitation, and vascular or orthopedic disorders. Importantly, no clinical trials have yet examined its effects on liver diseases, indicating a clear gap in the existing evidence base and suggesting a promising direction for future research.

Although most available studies investigating fisetin have been conducted in animal or in vitro models, emerging human data provide additional insights into its pharmacological behavior. Clinical studies evaluating fisetin in the context of aging, inflammatory conditions, viral infections, and cancer suggest that the compound is generally well-tolerated; however, its low oral bioavailability remains a significant challenge. Fisetin undergoes extensive first-pass metabolism, resulting in rapid glucuronidation and sulfation. For this reason, recent research has focused on alternative routes of administration and advanced delivery systems. Nanoemulsions, solid lipid nanoparticles, liposomal encapsulation, and cyclodextrin complexes have all been shown to improve solubility, stability, and systemic exposure. These technologies may be particularly relevant for potential therapeutic applications in IFALD, where enhanced bioavailability and controlled delivery are essential. Future studies should compare these formulations directly in both preclinical and clinical settings to identify the most effective strategy for fisetin delivery.

## 8. Conclusions and Future Perspectives

The available evidence highlights fisetin as a promising natural compound with broad hepatoprotective activity. Its multifaceted effects—including anti-inflammatory, antioxidant, anti-fibrotic, lipid-regulating, and microbiota-modulating actions—have been demonstrated across diverse models of liver injury. These mechanisms are highly relevant to the complex and multifactorial pathogenesis of intestinal failure-associated liver disease (IFALD), a serious complication of prolonged parenteral nutrition. Fisetin modulates key molecular pathways such as NF-κB, Nrf2, AMPK, and SIRT1, thereby counteracting oxidative stress, suppressing pro-inflammatory mediators, improving lipid and bile acid homeostasis, and attenuating fibrotic remodeling. Moreover, its influence on gut microbiota composition may provide an additional benefit by supporting gut–liver axis homeostasis.

This review synthesizes current knowledge on fisetin’s molecular actions and contextualizes these mechanisms within IFALD pathophysiology, offering a comprehensive mechanistic and translational perspective. However, several limitations should be acknowledged. The available evidence is predominantly preclinical and derives from heterogeneous experimental models with substantial variability in dosing regimens, endpoints, and study design; importantly, no clinical trials have evaluated fisetin specifically in IFALD to date. In addition, as a narrative review, this work does not include a quantitative synthesis or a formal risk-of-bias assessment.

Despite these constraints, fisetin remains a biologically active and generally safe compound whose clinical utility is currently limited mainly by poor water solubility and low systemic bioavailability. Recent advances in drug delivery systems—including nanoformulations and liposomal encapsulation—may help overcome these barriers and facilitate more effective (including parenteral) administration. Future work should prioritize standardized experimental approaches, clinically relevant in vivo models that better reflect the clinical course of IFALD, and early-phase clinical studies to validate fisetin’s efficacy, safety, and optimal delivery strategies. Overall, fisetin warrants further investigation as a potential therapeutic agent for preventing and treating IFALD.

## Figures and Tables

**Table 1:** Summary of research on fisetin’s hepatoprotective properties.

Experimental Model	Hepatotoxic Agent	Daily Dose/Concentration; Duration/Route of Administration	Effect of Fisetin on Liver Function	Mechanism	Reference
C57BL/6 mice	Ethanol	5 mg/kg, 10 mg/kg body weight; 8 days/orally	ALT ↓, AST ↓	NF-κB ↓, HO-1 ↓, NQO1 ↑, SOD ↑, CAT ↑, pro-MMP-2 ↓, active MMP-2 ↓, MMP-9 ↓	[5]
C57BL/6J mice	Ethanol	10 mg/kg body weight; 4 weeks/orally	ALT ↓, AST ↓, TG ↓, FFA ↓	ROS ↓, p-AMPK ↑	[6]
C57BL/6 mice/L-02 cell line	APAP	20 mg/kg, 80 mg/kg body weight; 7 days/orally 5 μM, 50 μM; 48 h	ALT ↓, AST ↓	ROS ↓, GSH ↑, IL-1β ↓, IL-18 ↓	[17]
Albino wistar rats	APAP	25 mg/kg, 50 mg/kg body weight; orally by gastric gavage	ALT ↓, AST ↓, ALP ↓, MDA ↓	SOD ↑, GSH ↑, TNF-α ↓, NF-κB ↓	[31]
C57 mice/L-02 cell line	APAP	10 mg/kg, 20 mg/kg, 40 mg/kg body weight; 7 days/orally by gastric gavage 50 μM; 24 h	ALT ↓, AST ↓, MDA ↓,	GSH ↑, ROS ↓, GPX1 ↑, CAT ↑, SOD1 ↑, SOD2 ↑	[32]
Wistar rats	Methotrexate	50 mg/kg body weight; 10 days/intraperitoneal	MDA ↓, ALT ↓	TGF-β ↓, SIRT1 ↑	[15]
Male albino rats	Fluoxetine	100 mg/kg body weight; 3 weeks/orally by orogastric tube	ALT ↓, AST ↓, MDA ↓	SOD ↑, GSH ↑	[33]
Albino Wistar rats	Thioacetamide	50 mg/kg, 100 mg/kg; 6 weeks/orally by gastric gavage	ALT ↓, AST ↓, ALP ↓ total bilirubin ↓, MDA ↓	IL-6 ↓, TNF-α ↓, GSH ↑, TGF-β ↓, MMP-9 ↑, α-SMA ↓	[16]
Male albino rats	Arsenic	2.5 mg/kg body weight, 30 days/orally	ALT ↓, AST ↓, ALP ↓	CAT ↑, SOD ↑, GSR ↑, GSH ↑, TNF-α ↓, NF-κB ↓, IL-6 ↓, IL-1β ↓, COX-2 ↓	[34]
Male C57BL/6J mice	High-fat diet	0.02% *w*/*w*; 16 weeks/orally	FA ↓, TG ↓	HMGCR ↓, ACAT ↓, PPARγ ↓, SREBP ↓, SCD1 ↓, β-oxydation ↑ TNF-α ↓, IL-6 ↓, MMP-2 ↓, MMP-9 ↓, TLR4 ↓	[11]
Male C57BL/6 mice	High-fat, high-sucrose diet	HFSD with 0.2% co-amorphous fisetin; 3 months/orally	ALT ↓, LDL ↓, TC ↓, liver cholesterol levels ↓	*Akkermansia muciniphila ↑, Butyricicoccus pullicaecorum ↑, Bifidobacterium breve* ↑	[35]
C57BL/6 mice/ NCTC1469 cell line	High-fat diet/ palmitate	20 mg/kg, 40 mg/kg, 80 mg/kg body weight; 20 weeks/orally by gastric gavage 10, 20, 40 μM; 24 h	AST ↓, ALT ↓, TG ↓, TC ↓, LDL ↓, HDL ↓	TNF-α ↓, IL-1β ↓, IL-6 ↓, PPAR-α ↑, SCD1 ↓, SREBP ↓	[12]
C57BL/6 mice/FL83B cell line	High-fat diet/ oleic acid	20 mg/kg body weight, twice a week for 10 weeks/intraperitoneal injection 0–100 µM/24 h	TG ↓, FFA ↓, lipolysis ↑, β-oxidation ↑	SREB-1C ↓, FAS ↑, PPARα ↑, PPARγ ↓, SIRT1 ↑, AMPKα ↑, p-AMPKα ↑	[28]
Albino rats	High-fat/ high-sucrose diet	10 mg/kg body weight 12 weeks/orally	TC ↓, LDL ↓, AST ↓, ALT ↓	PARP-1 ↓, ROS ↓, HNF4-α ↑	[14]
C57BL/6 mice/ NCTC1469, AML12 cell lines	High-fat diet/ palmitate	20 mg/kg, 40 mg/kg, 80 mg/kg; 20 weeks/orally by gastric gavage 10, 20 and 40 µM/24 h	ALT ↓, AST ↓, TC ↓, TG ↓	PPAR-α ↑, SREB-1C ↓, SCD1 ↓, IL-1β ↓, TNF-α ↓, IL-6 ↓, IL-18 ↓, NF-κB ↓, p-NF-κB ↓	[13]
C57BL/6N mice/L02, AML12 cell lines	High-fat diet/ palmitate	80 mg/kg 8 weeks/orally by gastric gavage 20 µM/24 h	ALT ↓, AST ↓, TC ↓, TG ↓, MDA ↓	TNF-α ↓, IL-6 ↓, ROS ↓, SOD ↑, CAT ↑, GSH ↑, HO-1 ↑, NQO1 ↑, GCLM ↑, Nrf2 ↑, FAS ↓, SCD1 ↓, PPARγ ↓	[36]
C57BL/6 mice/ HEK293T cell lines	Lipopolysaccharide/ cecal ligation and puncture (CLP)	10 mg/kg	ALT ↓, AST ↓,	NF-κB ↓	[37]
C57BL/6J mice (Mdr2^−/−^ mice/primary sclerosing cholangitis)	None	100 mg/kg body weight 7 days for 2 months/orally by gastric gavage		TNF-α ↓, IL-1ß ↓, IL-6 ↓	[38]
BALB/c mice	Lipopolysaccharide, galactosamine	3.5 mg/kg; for 78h every 6 h/intravenous	ALT ↓	nuclear Nrf2 ↑, NF-κB ↓, NLRP3 ↓, IL-1ß ↓	[39]
Male BALB/c mice/ Kupffer cells, U937, AML-12, NMu3Li, FL83B, NCTC1469 cell lines	Listeria monocytogenes	20 mg/kg, 40 mg/kg, 80 mg/kg body weight, 3 times a week for 4 weeks, intraperitoneal injection 10/20/40 µg/mL	ALT ↓, AST ↓, MDA ↓,	TNF-α ↓, IL-6 ↓, IL-18 ↓, IL-1β ↓, IKKα ↓, IKKß ↓, IκBα ↓, NLRP3 ↓, SOD ↑, GST ↑, CAT ↑, Nrf2 ↑	[40]
Columbia Cross female sheep	None	100 mg/kg body weight; two consecutive days for 8 weeks/intravenous		SOD1 ↓, CAT ↓, NLRP3 ↓	[41]
C57BL/6J mice/ RAW264.7 macrophages	Hepatic ischemia–reperfusion (I/R)	5 mg/kg, 10 mg/kg 20 mg/kg body weight; intraperitoneally 2.5, 5, 10 µmol/L	ALT ↓, AST ↓,	IL-1ß ↓, IL-18 ↓, TNF-α ↓, AMPK ↑, p-AMPK ↑, NLRP3 ↓,	[42]
C57BL/6 mice/AML-12 hepatocytes	Hepatic ischemia–reperfusion (I/R)	25 mg/kg, 50 mg/kg body weight; 10 mmol/L, 20 mmol/L	ALT ↓, AST ↓	ROS ↓, SOD ↑, cytoplasmic Nrf2 ↓, nuclear Nrf2 ↑, HO-1 ↑	[43]

Abbreviations: α-SMA, alpha-smooth muscle actin; ACAT, acylotransferaza acylo-CoA; ALP, alkaline phosphatase; ALT, alanine transaminase; AMPKα, AMP-activated protein kinase; APAP, acetaminophen; AST, aspartate transaminase; CAT, catalase; COX-2, cyclooxygenase-2; FAS, fatty acid synthase; GPX1, glutathione peroxidase 1; GSH, glutathione; GSR, glutathione reductase; HDL, high-density lipoprotein; HMGCR, HMG-CoA reductase; HNF4α, hepatocyte nuclear factor 4 alfa; HO-1, heme-oxygenase 1, IκBα, inhibitor of κB alpha; IKKα, IκB kinase alfa; IKKß, IκB kinase beta; IL-1β, interleukin-1β; IL-6, interleukin-6; IL-18, interleukin-18; LDL, low-density lipoprotein; MDA, 3,4-methylenedioxyamphetamine; MMP-2, matrix metalloproteinase-2; MMP-9, matrix metalloproteinase-9; NF-κB, nuclear factor kappa B; NLRP3, NLR family pyrin domain containing 3; NQO1, NAD(P)H: quinone oxidoreductase 1; Nrf2, nuclear factor erythroid 2–related factor 2; p-AMPKα, AMP-activated protein kinase; PARP-1, poly-(ADP-ribose)-polymerase-1; PPARα, peroxisome proliferator-activated receptor alfa; PPARγ, peroxisome proliferator-activated receptor gamma; ROS, reactive oxygen species; SREBP, sterol regulatory element-binding protein; SOD1, superoxide dismutase 1; SOD2, superoxide dismutase 2; SIRT-1, sirtuin 1; SCD1, stearyl-coenzyme A desaturase 1;TC, total cholesterol; TG, triglycerides; TGF-β, transforming growth factor beta; TLR4, Toll-like receptor 4; TNF-α, tumor necrosis factor; ↑ increase; ↓ decrease.

**Table 2:** Factors leading to the development of IFALD.

Factor	Mechanism	Ref.
Phytosterols in Parenteral Nutrition	inhibits FXR in the liver, resulting in increased bile acid and triglyceride production.	[45]
Lipopolysaccharide (LPS) from CLABSI and gut dysbiosis	activates the NF-κB pathway. promotes pro-inflammatory cytokine release. impairs bile transporter function, reducing bile acid efflux from the liver.	[46]
Omega-6 fatty acids in Parenteral Nutrition	serve as precursors for pro-inflammatory eicosanoids. activate the TLR4 pathway, mimicking LPS-induced inflammation.	[47]
Oxidative Stress	inflammation increases ROS production, causes mitochondrial dysfunction, and endoplasmic reticulum (ER) stress. omega-6-rich lipid emulsions: ○ promote lipid peroxidation and hepatocyte damage. ○ downregulate antioxidant defense genes reduced secretion of antioxidant enzymes (e.g., GSH, SOD, catalase) disrupts redox balance.	[48,49]
Lipid and Bile Acid Metabolism Disruption	leads to hepatic lipid accumulation and liver steatosis.	[50]

## Data Availability

No new data were created or analyzed in this study. Data sharing is not applicable to this article.

## Abbreviations

The following abbreviations are used in this manuscript:

AMPK	AMP-activated protein kinase
COVID-19	coronavirus disease 2019
CXCL-2	chemokine (C-X-C motif) ligand 2
ERK1/2	extracellular signal-regulated kinase 1/2
GPX1	glutathione peroxidase 1
GSase	galactosylceramide sulfotransferase
GSH	glutathione
GSR	glutathione reductase
Gstm1	glutathione S-transferase Mu 1
Gstp1	glutathione S-transferase Pi 1
HO-1	heme-oxygenase 1
HSF1	heat shock factor protein 1
FAS	fatty acid synthase
FGFR4	fibroblast growth factor receptor 4
FGFR19	fibroblast growth factor 19
FXR	farnesoid X receptor
IFALD	Intestinal Failure-Associated Liver Disease
IL-1β	interleukin-1β
IL-6	interleukin-6
IL-18	interleukin-18
LPL	lipoprotein lipase
LPS	lipopolysaccharide
MMP-2	matrix metalloproteinase-2
MMP-9	matrix metalloproteinase-9
NAFLD	non-alcoholic fatty liver disease
NF-κB	nuclear factor kappa B
NLRP3	NLR family pyrin domain containing 3
NQO1	NAD(P)H Quinone oxidoreductase 1
Nrf2	nuclear factor erythroid 2–related factor 2
p53	tumor protein p53
PAI-1	plasminogen activator inhibitor-1
PI3K	phosphoinositide 3-kinase
PN	parenteral nutrition
PPARα	peroxisome proliferator-activated receptor alfa
ROS	reactive oxygen species
SASP	senescent associated secretory phenotypes
SCD1	stearyl-coenzyme A desaturase 1
SIRT1	sirtuin 1
SOD2	superoxide dismutase 2
SREBP-1c	sterol regulatory element-binding protein 1
TGF-β	transforming growth factor beta 1
TLR4	Toll-like receptor 4
TNF-α	tumor necrosis factor alfa
TPN	total parenteral nutrition
